# Association of *ALDH3B2* gene polymorphism and risk factors with susceptibility of esophageal squamous cell carcinoma in a Chinese population: a case-control study involving 2,358 subjects

**DOI:** 10.18632/oncotarget.22656

**Published:** 2017-11-24

**Authors:** Jun Yin, Weifeng Tang, Tao Long, Huiwen Pan, Jianchao Liu, Lu Lv, Chao Liu, Yijun Shi, Jingfeng Zhu, Yangyong Sun, Aizhong Shao, Qiang Zhou, Zhengbing Ren, Guowen Ding, Suocheng Chen, Yan Liu, Jun Yao, Hao Ding, Yulan Yan, Haiyong Gu, Cheng Qian, Liming Wang, Qun Wang, Lijie Tan

**Affiliations:** ^1^ Department of Cardiothoracic Surgery, Affiliated People's Hospital of Jiangsu University, Zhenjiang, Jiangsu, 212002, China; ^2^ Department of Thoracic Surgery, Zhongshan Hospital of Fudan University, Shanghai, 200032, China; ^3^ Genesky Biotechnologies Inc., Shanghai, 201315, China; ^4^ Department of Gastroenterology, Affiliated People's Hospital of Jiangsu University, Zhenjiang, Jiangsu, 212002, China; ^5^ Department of Respirology, Affiliated People's Hospital of Jiangsu University, Zhenjiang, Jiangsu, 212002, China; ^6^ Department of Thoracic Surgery, Shanghai Chest Hospital, Shanghai Jiaotong University, Shanghai, 200030, China; ^7^ Cancer Institute, Department of Chemotherapy, Affiliated People's Hospital of Jiangsu University, Zhenjiang, Jiangsu, 212002, China

**Keywords:** ALDH3B2, polymorphisms, risk factors, esophageal squamous cell carcinoma

## Abstract

**Background:**

Esophageal cancer (EC) is the sixth leading cause of cancer-associated death worldwide. The interaction of environmental risk factors and genetic factors might contribute to the carcinogenesis of EC synergistically.

**Results:**

All seven single locus polymorphisms of ALDH3B2 were not associated with risk of ESCC as evaluated by allelic, dominant, co-dominant, recessive and Cochran-Armitage trend tests. Stratified analyses showed these SNPs were not correlated with the susceptibility of ESCC according to different age, gender, cigarette smoking and alcohol drinking status. None of the major haplotypes were related with ESCC susceptibility.

**Materials and Methods:**

We conducted a hospital-based case–control study to evaluate the combined effects of environmental risk factors and the single nucleotide polymorphisms (SNPs) of ALDH3B2 gene on the development of esophageal squamous carcinoma (ESCC). A total of 1043 ESCC cases and 1315 controls were recruited for this study. Seven ALDH3B2 SNPs and four environmental factors were selected as independent variables. ALDH3B2 SNPs were determined by ligation detection reaction method.

**Conclusions:**

Our study suggested that ALDH3B2 rs34589365, rs3741172, rs4646823, rs78402723, rs7947978, rs866907 and rs9787887 polymorphisms were not implicated with altered susceptibility of ESCC according to different age, gender, cigarette smoking and alcohol drinking status. Yet this conclusion needs to be verified in larger studies among different ethnic populations with validation design, the biological function of these SNPs in carcinogenesis are subject to further investigation.

## INTRODUCTION

Esophageal cancer ranks the ninth most common cancer and the sixth most common cause of cancer death worldwide [1]. Despite remarkable advances in the therapeutic strategy, extensive treatment may be associated with a noticeable decline in health-related quality of life and yet a poor prognosis [2]. Approximately 70% of global esophageal cancer cases occur in China, with esophageal squamous cell carcinoma (ESCC) being the histopathological form in the vast majority of cases (> 90%) [3]. Alcohol consumption [4, 5], tobacco use [4, 6], poor oral hygiene, low socioeconomic status and nutritional deficiencies have been reported risk factors for esophageal cancer [7–10]. The fact that only a subset of cohort that are exposed to the risk factors eventually develop esophageal cancer suggested a critical role of genetic factors, including single nucleotide polymorphisms (SNPs), in the esophageal carcinogenesis.

Among all risk factors, others and we have repeatedly verified the indisputable role of alcohol consumption in the ESCC carcinogenesis [11, 12]. Extensive evaluations and reviews of alcohol-related cancers have been published in the Monographs of the International Agency of Research on Cancer (IARC) [13, 14], with most convincing evidence (Group I) for alcohol-drinking-related cancers is targeted on esophagus and some other organs [15]. Specific enzymes, whose activity and expression are influenced by genetic polymorphisms, regulate the metabolism of alcohol [11]. In humans, alcohol is primarily metabolized by two major groups of enzymes termed alcohol dehydrogenases (ADHs) and aldehyde dehydrogenases (ALDHs) [16]. In the cytosol of hepatocytes, ADHs catalyse the oxidation of ethanol to acetaldehyde, which is further oxidized to acetate by ALDHs in the mitochondria [11]. Although alcohol is not a carcinogen *per se*, its metabolite acetaldehyde is a toxin and carcinogen that rapidly binds to protein and DNA. It has profound effects on carcinogenesis by forming with DNA carcinogenic DNA adducts, by inhibiting DNA repair and by regulating DNA methylation. Acetaldehyde is degradated by ALDHs, which renders ALDHs a pivotal role in the carcinogenesis. Indeed, high ALDH1 expression predicts unfavorable outcomes in patients with ESCC [17]. Individuals with *ALDH2* Lys allele possess a higher risk of esophageal cancer, in correlation with a higher concentration of blood acetaldehyde after drinking alcohol [18]. There is a strong association between *ALDH2* Glu487Lys polymorphism and the risk of esophageal cancer [19]. *ALDH2* rs671 [20] and rs886205 [21] polymorphisms have also been demonstrated to correlate with ESCC, respectively.

Similarly, the aldehyde dehydrogenase 3 family member B2 (ALDH3B2) is also a key member of ALDH family. Originally identified as ALDH8, ALDH3B2 encodes a member of the aldehyde dehydrogenase family, a group of isozymes that may play a major role in the detoxification of aldehydes generated by alcohol metabolism and lipid peroxidation. As compared with ALDH1/2, little is known on ALDH3 family (including ALDH3B2) with respect to their roles in carcinogenesis. The association between ALDH3B2 polymorphisms and ESCC has not been investigated. Hence, in this hospital-based case-control study, we performed genotyping analyses of the seven SNPs in 1043 ESCC cases and 1315 controls in a Chinese population.

## RESULTS

### Characteristics of the study subjects

The characteristics of the study subjects, including the demographics and environmental risk factors, are presented in Table 1. The controls and cases were well matched in age and gender (χ^2^ test, *p* = 0.121 and 0.880, respectively). However, the cigarette-smoking rate (43.53% vs.26.70%, *p* < 0.001) and alcohol drinking rate (31.54% vs.7.07%, *p* < 0.001) were both significantly higher in the ESCC cases.

**Table 1:** Distribution of selected demographic variables and risk factors in ESCC cases and controls

Variable	Cases (*n* = 1043)		Controls (*n* = 1315)		*p*
	*n*	%	*n*	%	
**Age (years) mean ± SD**	63.07(± 7.27)		62.88 (± 9.74)		0.607
**Age (years)**					
< 63	471	45.16	636	48.37	
≥ 63	572	54.84	679	51.63	0.121
**Sex**					
Male	758	72.67	952	72.40	
Female	285	27.33	363	27.63	0.880
**Tobacco use**					
Never	589	56.47	964	73.30	
Ever	454	43.53	351	26.70	**< 0.001**
**Alcohol use**					
Never	714	68.46	1222	92.93	
Ever	329	31.54	93	7.07	**< 0.001**

^a^ Two-sided *χ*^2^ test and student t test; Bold values are statistically significant (*p* < 0.05).

As shown in Table 2, the genotyping successful rates were all beyond 98.81%. In the control subjects, the genotype frequencies for the seven polymorphisms reached Hardy-Weinberg equilibrium (*p*-value for HWE, all *p* > 0.05, Table 2).

**Table 2:** Primary information for *ALDH3B2* rs34589365, rs3741172, rs4646823, rs78402723, rs7947978, rs866907, rs9787887 polymorphisms

Genotyped SNPs	rs34589365	rs3741172	rs4646823	rs78402723	rs7947978	rs866907	rs9787887
Ancestral Allele	A	G	G	G	C	A	G
Chromosome	11	11	11	11	11	11	11
Gene (ID)	ALDH3B2(222)	ALDH3B2(222)	ALDH3B2(222)	ALDH3B2(222)	ALDH3B2(222)	ALDH3B2(222)	ALDH3B2(222)
Function	intron-variant	reference, synonymous-codon	utr-variant-5-prime	utr-variant-3-prime	intron-variant, utr-variant-5-prime	utr-variant-3-prime	intron-variant
Chr Pos (Genome Build 38.p7)	67668505	67663227	67666945	67663118	67674572	67663011	67673240
Regulome DB Score^a^	5	5	4	4	4	4	5
TFBS^b^	Y	--	Y	--	--	--	--
nsSNP	--	--	--	--	--	--	--
MAF^c^ for Chinese in database	0.183	--	0.098	0.058	0.098	0.128	--
MAF in our controls (n = 1315)	0.126	0.084	0.105	0.084	0.105	0.105	0.335
*p* value for HWE^d^ test in our controls	0.110	0.784	0.294	0.808	0.279	0.294	0.258
Genotyping method^e^	LDR	LDR	LDR	LDR	LDR	LDR	LDR
% Genotyping value	99.02%	99.02%	99.02%	98.98%	99.02%	99.02%	98.81%

^a^http://www.regulomedb.org/;

^b^TFBS: Transcription Factor Binding Site (https://snpinfo.niehs.nih.gov/cgi-bin/snpinfo/snpfunc.cgi);

^c^MAF: minor allele frequency;

^d^HWE: Hardy–Weinberg equilibrium;

^e^LDR: ligation detection reaction.

### Associations between risk of ESCC and seven polymorphisms

As shown in Table 3, the single locus analyses showed no statistically significant difference in genotype frequencies of seven SNPs between the cases and controls (*p* > 0.05). As assessed by the allelic, dominant, co-dominant, recessive and Cochran-Armitage trend tests, there are no correlations between these seven polymorphism sites with the risk of ESCC (Table 3).

**Table 3:** Main effects of *ALDH3B2* SNPs on ESCC risk

Locus	Geno-type	Case	Control	Co-dominant	Dominant	Recessive	Cochran-Armitage trend	Allelic test
				χ^2^ *P*	χ^2^ *P*	χ^2^ *P*	χ^2^ *P*	χ^2^ *P*
rs34589365 *A/G*	*AA*	13 (1.27%)	27 (2.06%)					
	*AG*	228 (22.24%)	275 (20.99%)	2.544; 0.2803	0.0678; 0.7945	2.147; 0.1429	0.0290; 0.8649	0.0294; 0.8639
	*GG*	784 (76.49%)	1008 (76.95%)					
rs3741172 *G/A*	*GG*	839 (81.85%)	1100 (83.97%)					
	*AG*	176 (17.17%)	200 (15.27%)	1.906; 0.3855	1.828; 0.1764	0.3051; 0.5807	1.906; 0.1674	1.92; 0.1658
	*AA*	10 (0.98%)	10 (0.76%)					
rs4646823 *G/T*	*GG*	15 (1.46%)	18 (1.37%)					
	*GT*	202 (19.71%)	239 (18.24%)	0.8581; 0.6511	0.8566; 0.3547	0.0330; 0.8559	0.7819; 0.3766	0.8008; 0.3709
	*TT*	808 (78.83%)	1053 (80.38%)					
rs78402723 *G/A*	*GG*	849 (82.91%)	1099 (83.89%)					
	*AG*	166 (16.21%)	201 (15.34%)	0.4358; 0.8042	0.4023; 0.5259	0.0950; 0.7579	0.4343; 0.5099	0.4377; 0.5082
	*AA*	9 (0.88%)	10 (0.76%)					
rs7947978 *C/A*	*CC*	804 (78.44%)	1054 (80.46%)					
	*CA*	209 (20.39%)	238 (18.17%)	1.963; 0.3747	1.442; 0.2298	0.1874; 0.6651	0.9688; 0.325	0.9794; 0.3223
	*AA*	12 (1.17%)	18 (1.37%)					
rs866907 *A/G*	*AA*	12 (1.17%)	18 (1.37%)					
	*AG*	204 (19.90%)	239 (18.24%)	1.171; 0.5568	0.7535; 0.3854	0.1874; 0.6651	0.4626; 0.4964	0.4686; 0.4936
	*GG*	809 (78.93%)	1053 (80.38%)					
rs9787887 *G/A*	*GG*	129 (12.63%)	156 (11.92%)					
	*AG*	454 (44.47%)	565 (43.16%)	0.9959; 0.6078	0.9504; 0.3296	0.2748; 0.6001	0.9316; 0.3344	0.9567; 0.328
	*AA*	438 (42.90%)	588 (44.92%)					

### Stratification analyses on seven polymorphisms and risk of ESCC

To further evaluate the effects of *ALDH3B2* rs34589365, rs3741172, rs4646823, rs78402723, rs7947978, rs866907 and rs9787887 on ESCC risk with different gender, age, smoking and alcohol drinking status, stratification analyses were performed as demonstrated in the Tables 4–10.

**Table 4:** Stratified analyses between *ALDH3B2* rs34589365 polymorphism and ESCC risk by sex, age, smoking status and alcohol consumption

Variable	(case/control) ^a^				Adjusted OR ^b^ (95%CI); *p*				
	AA	GG	AG	AG+GG	AA	GG	AG	AG+GG	GG vs. (AA+AG)
Sex									
Male	12/19	554/717	179/212	733/929	1.00	0.817 (0.39–1.70); *p*:0.588	0.748 (0.35–1.58); *p*:0.446	0.800 (0.39–1.66); *p*:0.547	0.934 (0.75–1.17); *p*:0.549
Female	1/8	230/291	49/63	279/354	1.00	1.158 (0.02–1.27); *p*:0.085	0.161 (0.02–1.33); *p*:0.079	0.159 (0.02–1.28); *p*:0.085	1.122 (0.75–1.68); *p*:0.573
Age									
< 63	5/14	347/478	114/140	461/618	1.00	0.492 (0.18–1.38); *p*:0.239	0.439 (0.15–1.25); *p*:0.151	0.479 (0.17–1.34); *p*:0.168	0.939 (0.71–1.24); *p*:0.658
≥ 63	8/13	437/530	114/135	551/665	1.00	0.746 (0.31–1.82); *p*:0.659	0.729 (0.29–1.82); *p*:0.497	0.746 (0.31–1.82); *p*:0.518	1.000 (0.76–1.31); *p*:0.999
Smoking status									
Never	8/21	449/743	117/196	566/939	1.00	0.630 (0.28–1.44); *p*:0.268	0.638 (0.27–1.49); *p*:0.295	0.632 (0.28–1.44); *p*:0.269	1.049 (0.82–1.35); *p*:0.706
Ever	5/6	335/265	111/79	446/344	1.00	0.659 (0.20–2.18); *p*:0.550	0.593 (0.18–2.01); *p*:0.533	0.643 (0.20–2.12); *p*:0.547	0.926 (0.67–1.28); *p*:0.642
Alcohol consumption									
Never	10/26	555/945	138/246	693/1191	1.00	0.655 (0.31–1.37); *p*:0.257	0.686 (0.32–1.46); *p*:0.327	0.661 (0.32–1.38); *p*:0.267	1.079 (0.86–1.35); *p*:0.508
Ever	3/1	229/63	90/29	319/92	1.00	0.825 (0.08–8.07); *p*:1.000	0.967 (0.10–9.66); *p*:1.000	0.865 (0.09–8.42); *p*:1.000	1.173 (0.71–1.93); *p*:0.530

^a^The genotyping success rate was 99.02% for rs34589365; ^b^Adjusted for age, sex, smoking status and alcohol consumption (besides stratified factors accordingly) in a logistic regression model; Fisher exact test was performed when *n* ≤ 5; Bonferroni correction was performed to correct the *p* value, bold values are statistically significant (*p* < 0.0125).

**Table 5:** Stratified analyses between *ALDH3B2* rs3741172 polymorphism and ESCC risk by sex, age, smoking status and alcohol consumption

Variable	(case/control)^a^				Adjusted OR b (95%CI); *p*				
	GG	AA	AG	AA+AG	GG	AA	AG	AA+AG	AA vs. (GG+AG)
Sex									
Male	622/798	5/7	118/143	123/150	1.00	1.091 (0.35–3.46); *p*:1.000	0.945 (0.72–1.23); *p*:0.674	0.951 (0.73–1.23); *p*:0.703	0.908 (0.29–2.87); *p*:1.000
Female	217/302	5/3	58/57	63/60	1.00	0.431 (0.10–1.82); *p*:0.291	0.706 (0.47–1.06); *p*:0.091	0.684 (0.46–1.02); *p*:0.059	2.176 (0.52–9.18); *p*:0.305
Age									
< 63	387/527	4/8	75/97	79/105	1.00	1.469 (0.44–4.91); *p*:0.770	0.950 (0.68–1.32); *p*:0.759	0.976 (0.71–1.34); *p*:0.882	0.675 (0.20–2.26); *p*:0.573
≥ 63	452/573	6/2	101/103	107/105	1.00	0.263 (0.05–1.31); *p*:0.149	0.804 (0.60–1.09); *p*:0.156	0.774 (0.58–1.04); *p*:0.090	3.667 (0.74–18.24); *p*:0.151
Smoking status									
Never	463/811	6/7	105/142	111/149	1.00	0.666 (0.22–1.99); *p*:0.465	0.772 (0.59–1.02); *p*:0.067	0.766 (0.58–1.01); *p*:0.054	1.438 (0.48–4.30); *p*:0.513
Ever	376/289	4/3	71/58	75/61	1.00	0.976 (0.22–4.39); *p*:1.000	1.063 (0.73–1.55); *p*:0.753	1.058 (0.73–1.53); *p*:0.765	1.035 (0.23–4.66); *p*:1.000
Alcohol consumption									
Never	571/1024	7/9	125/184	132/193	1.00	0.717 (0.27–1.94); *p*:0.509	0.821 (0.64–1.05); *p*:0.120	0.815 (0.64–1.04); *p*:0.100	1.350 (0.50–3.64); *p*:0.552
Ever	268/76	3/1	51/16	54/17	1.00	1.175 (0.12–11.46); *p*:1.000	1.106 (0.60–2.05); *p*:0.748	1.110 (0.61–2.03); *p*:0.733	0.865 (0.09–8.42); *p*:1.000

^a^ The genotyping success rate was 99.02% for rs3741172; ^b^ Adjusted for age, sex, smoking status and alcohol consumption (besides stratified factors accordingly) in a logistic regression model; Fisher exact test was performed when n ≤ 5; Bonferroni correction was performed to correct the *p* value, bold values are statistically significant (*p* < 0.0125).

**Table 6:** Stratified analyses between *ALDH3B2* rs4646823 polymorphism and ESCC risk by sex, age, smoking status and alcohol consumption

Variable	(case/control) a				Adjusted OR b (95%CI); p				
	GG	TT	GT	TT+GT	GG	TT	GT	TT+GT	TT vs. (GG+GT)
Sex									
Male	10/12	598/762	137/174	735/936	1.00	1.062 (0.46–2.48); *p*:0.889	1.058 (0.44–2.52); *p*:0.898	1.061 (0.46–2.47); *p*:0.890	0.993 (0.78–1.26); *p*:0.954
Female	5/6	210/291	65/65	275/356	1.00	1.155 (0.35–3.83); *p*:1.000	0.833 (0.24–2.88); *p*:1.000	1.079 (0.33–3.57); *p*:1.000	0.732 (0.50–1.07); *p*:0.102
Age									
< 63	5/11	373/506	88/115	461/621	1.00	0.617 (0.21–1.79); *p*:0.450	0.594 (0.20–1.77); *p*:0.436	0.612 (0.21–1.77); *p*:0.450	0.999 (0.74–1.35); *p*:0.993
≥ 63	10/7	435/547	114/124	549/671	1.00	1.796 (0.68–4.76); *p*:0.232	1.554 (0.57–4.22); *p*:0.384	1.746 (0.66–4.62); *p*:0.255	0.840 (0.64–1.01); *p*:0.216
Smoking status									
Never	8/14	450/777	116/169	566/946	1.00	0.987 (0.41–2.37); *p*:0.976	0.833 (0.34–2.05); *p*:0.689	0.955 (0.40–2.29); *p*:0.918	0.855 (0.66–1.04); *p*:0.229
Ever	7/4	358/276	86/70	444/346	1.00	1.349 (0.39–4.66); *p*:0.764	1.424 (0.40–5.06); *p*:0.756	1.364 (0.40–4.70); *p*:0.764	1.032 (0.73–1.46); *p*:0.857
Alcohol consumption									
Never	10/17	556/978	137/222	693/1200	1.00	1.035 (0.47–2.28); *p*:0.932	0.953 (0.42–2.14); *p*:0.908	1.019 (0.46–2.24); *p*:0.963	0.924 (0.73–1.16); *p*:0.503
Ever	5/1	252/75	65/17	317/92	1.00	1.488 (0.17–12.94); *p*:1.000	1.308 (0.14–11.95); *p*:1.000	1.451 (0.17–12.58); *p*:1.000	0.864 (0.48–1.54); *p*:0.620

^a^ The genotyping success rate was 99.02% for rs4646823; ^b^ Adjusted for age, sex, smoking status and alcohol consumption (besides stratified factors accordingly) in a logistic regression model; Fisher exact test was performed when *n* ≤ 5; Bonferroni correction was performed to correct the *p* value, bold values are statistically significant (*p* < 0.0125).

**Table 7:** Stratified analyses between *ALDH3B2* rs78402723 polymorphism and ESCC risk by sex, age, smoking status and alcohol consumption

Variable	(case/control)^a^				Adjusted OR^b^ (95%CI); *p*				
	GG	AA	AG	AA+AG	GG	AA	AG	AA+AG	AA vs. (GG+AG)
Sex									
Male	629/798	4/7	111/143	115/150	1.00	1.379 (0.40–4.73); *p*:0.764	0.985 (0.75–1.29); *p*:0.911	0.973 (0.75–1.27); *p*:0.837	0.727 (0.21–2.49); *p*:0.764
Female	220/301	5/3	55/58	60/61	1.00	0.439 (0.10–1.85); *p*:0.294	0.771 (0.51–1.16); *p*:0.210	0.743 (0.50–1.11); *p*:0.141	2.176 (0.52–9.18); *p*:0.305
Age									
< 63	392/526	4/8	70/98	74/106	1.00	1.490 (0.45–4.99); *p*:0.572	1.043 (0.75–1.46); *p*:0.803	1.068 (0.77–1.48); *p*:0.693	0.675 (0.20–2.26); *p*:0.573
≥ 63	457/573	5/2	96/103	101/105	1.00	0.319 (0.06–1.65); *p*:0.252	0.856 (0.63–1.16); *p*:0.315	0.829 (0.62–1.12); *p*:0.220	3.056 (0.59–15.81); *p*:0.255
Smoking status									
Never	467/810	6/7	101/143	107/150	1.00	0.673 (0.23–2.01); *p*:0.476	0.816 (0.62–1.08); *p*:0.154	0.808 (0.62–1.06); *p*:0.126	1.438 (0.48–4.30); *p*:0.513
Ever	382/289	3/3	65/58	68/61	1.00	1.322 (0.27–6.60); *p*:1.000	1.179 (0.80–1.73); *p*:0.401	1.186 (0.81–1.73); *p*:0.377	0.776 (0.16–3.87); *p*:1.000
Alcohol consumption									
Never	578/1023	7/9	118/185	125/194	1.00	0.726 (0.27–1.96); *p*:0.526	0.886 (0.69–1.14); *p*:0.346	0.877 (0.69–1.12); *p*:0.297	1.350 (0.50–3.64); *p*:0.552
Ever	271/76	2/1	48/16	50/17	1.00	1.783 (0.16–19.93); *p*:0.527	1.189 (0.64–2.21); *p*:0.585	1.212 (0.66–2.22); *p*:0.533	0.577 (0.05–6.43); *p*:0.535

^a^The genotyping success rate was 98.98% for rs78402723; ^b^Adjusted for age, sex, smoking status and alcohol consumption (besides stratified factors accordingly) in a logistic regression model; Fisher exact test was performed when *n* ≤ 5; Bonferroni correction was performed to correct the *p* value, bold values are statistically significant (*p* < 0.0125).

**Table 8:** Stratified analyses between *ALDH3B2* rs7947978 polymorphism and ESCC risk by sex, age, smoking status and alcohol consumption

Variable	(case/control) ^a^				Adjusted OR^b^ (95%CI); *p*				
	CC	AA	CA	AA+CA	CC	AA	CA	AA+CA	AA vs. (CC+CA)
Sex									
Male	595/762	7/12	143/174	150/186	1.00	1.339 (0.52–3.42); *p*:0.541	0.950 (0.74–1.22); *p*:0.683	0.968 (0.76–1.23); *p*:0.792	0.740 (0.29–1.89); *p*:0.527
Female	209/292	5/6	66/64	71/70	1.00	0.859 (0.26–2.85); *p*:1.000	0.694 (0.47–1.02); *p*:0.064	0.706 (0.49–1.03); *p*:0.068	1.079 (0.33–3.57); *p*:1.000
Age									
< 63	372/507	4/11	90/114	94/125	1.00	2.018 (0.64–6.39); *p*:0.295	0.929 (0.68–1.26); *p*:0.640	0.976 (0.72–1.32); *p*:0.872	0.489 (0.16–1.55); *p*:0.295
≥ 63	432/547	8/7	119/124	127/131	1.00	0.691 (0.25–1.92); *p*:0.476	0.823 (0.62–1.09); *p*:0.174	0.815 (0.62–1.07); *p*:0.143	1.392 (0.50–3.86); *p*:0.524
Smoking status									
Never	447/778	7/14	120/168	127/182	1.00	1.149 (0.46–2.87); *p*:0.766	0.824 (0.62–1.05); *p*:0.102	0.823 (0.64–1.06); *p*:0.134	0.834 (0.34–2.08); *p*:0.697
Ever	357/276	5/4	89/70	94/74	1.00	1.035 (0.28–3.89); *p*:1.000	1.017 (0.72–1.44); *p*:0.923	1.018 (0.72–1.44); *p*:0.918	0.970 (0.26–3.64); *p*:1.000
Alcohol consumption									
Never	552/979	9/17	142/221	151/238	1.00	1.065 (0.47–2.41); *p*:0.879	0.878 (0.69–1.11); *p*:0.276	0.889 (0.71–1.12); *p*:0.313	0.915 (0.41–2.07); *p*:0.831
Ever	252/75	3/1	67/17	70/18	1.00	1.120 (0.12–10.93); *p*:1.000	0.853 (0.47–1.54); *p*:0.597	0.864 (0.48–1.54); *p*:0.620	0.865 (0.09–8.42); *p*:1.000

^a^ The genotyping success rate was 99.02% for rs7947978; ^b^ Adjusted for age, sex, smoking status and alcohol consumption (besides stratified factors accordingly) in a logistic regression model; Fisher exact test was performed when *n* ≤ 5; Bonferroni correction was performed to correct the *p* value, bold values are statistically significant (*p* < 0.0125).

**Table 9:** Stratified analyses between *ALDH3B2* rs866907 polymorphism and ESCC risk by sex, age, smoking status and alcohol consumption

Variable	(case/control)^a^				Adjusted OR^b^ (95% CI); *p*				
	AA	GG	AG	GG+AG	AA	GG	AG	GG+AG	GG vs. (AA+AG)
Sex									
Male	7/12	598/762	140/174	738/936	1.00	0.743 (0.29–1.90); *p*:0.534	0.725 (0.28–1.89); *p*:0.509	0.740 (0.29–1.89); *p*:0.527	0.993 (0.78–1.26); *p*:0.954
Female	5/6	211/291	64/65	275/356	1.00	1.149 (0.35–3.82); *p*:1.000	0.846 (0.25–2.91); *p*:1.000	1.079 (0.32–3.57); *p*:1.000	0.746 (0.51–1.09); *p*:0.126
Age									
< 63	4/11	373/506	89/115	462/621	1.00	0.493 (0.16–1.56); *p*:0.295	0.470 (0.15–1.53); *p*:0.281	0.489 (0.16–1.55); *p*:0.295	0.999 (0.74–1.35); *p*:0.993
≥ 63	8/7	436/547	115/124	551/671	1.00	1.434 (0.52–3.99); *p*:0.487	1.232 (0.43–3.51); *p*:0.695	1.392 (0.50–3.86); *p*:0.524	0.849 (0.64–1.12); *p*:0.245
Smoking status									
Never	7/14	451/777	116/169	567/946	1.00	0.861 (0.35–2.15); *p*:0.749	0.728 (0.29–1.86); *p*:0.506	0.834 (0.34–2.08); *p*:0.697	0.864 (0.67–1.12); *p*:0.262
Ever	5/4	358/276	88/70	446/346	1.00	0.964 (0.26–3.62); *p*:1.000	0.994 (0.26–3.84); *p*:1.000	0.970 (0.26–3.64); *p*:1.000	1.032 (0.73–1.46); *p*:0.857
Alcohol consumption									
Never	9/17	557/978	137/222	694/1200	1.00	0.930 (0.41–2.10); *p*:0.860	0.858 (0.37–1.98); *p*:0.719	0.915 (0.41–2.07); *p*:0.831	0.932 (0.74–1.17); *p*:0.551
Ever	3/1	252/75	67/17	319/92	1.00	0.893 (0.09–8.71); *p*:1.000	0.761 (0.07–7.78); *p*:1.000	0.865 (0.09–8.42); *p*:1.000	0.864 (0.48–1.54); *p*:0.620

^a^ The genotyping success rate was 99.02% for rs866907; ^b^ Adjusted for age, sex, smoking status and alcohol consumption (besides stratified factors accordingly) in a logistic regression model; Fisher exact test was performed when *n* ≤ 5; Bonferroni correction was performed to correct the *p* value, bold values are statistically significant (*p* < 0.0125).

**Table 10:** Stratified analyses between *ALDH3B2* rs9787887 polymorphism and ESCC risk by sex, age, smoking status and alcohol consumption

Variable	(case/control) ^a^				Adjusted OR^b^ (95%CI); *p*				
	**GG**	**AA**	**AG**	**AG+AA**	**GG**	**AA**	**AG**	**AG+AA**	**AA vs. (GG+AG)**
Sex									
Male	84/113	319/420	338/414	657/834	1.00	0.979 (0.71–1.35); *p*:0.894	0.911 (0.66–1.25); *p*:0.562	0.944 (0.70–1.27); *p*:0.705	0.949 (0.78–1.15); *p*:0.593
Female	45/43	119/168	116/151	235/319	1.00	1.477 (0.92–2.39); *p*:0.110	1.362 (0.84–2.21); *p*:0.209	1.421 (0.91–2.23); *p*:0.126	0.854 (0.62–1.17); *p*:0.323
Age									
< 63	63/83	203/287	197/262	400/549	1.00	1.158 (0.80–1.67); *p*:0.433	1.090 (0.75–1.58); *p*:0.649	1.124 (0.80–1.59); *p*:0.506	0.939 (0.74–1.20); *p*:0.606
≥ 63	66/73	235/301	257/303	492/604	1.00	1.158 (0.80–1.68); *p*:0.442	1.066 (0.74–1.55); *p*:0.737	1.110 (0.78–1.58); *p*:0.563	0.909 (0.73–1.14); *p*:0.408
Smoking status									
Never	76/107	245/430	253/422	498/852	1.00	1.247 (0.89–1.74); *p*:0.194	1.241 (0.89–1.73); *p*:0.202	1.215 (0.89–1.66); *p*:0.223	0.916 (0.74–1.13); *p*:0.411
Ever	53/49	193/158	201/143	394/301	1.00	0.885 (0.57–1.38); *p*:0.589	0.770 (0.49–1.20); *p*:0.246	0.826 (0.55–1.25); *p*:0.369	0.923 (0.70–1.22); *p*:0.579
Alcohol consumption									
Never	91/141	305/552	306/523	611/1075	1.00	1.168 (0.87–1.57); *p*:0.307	1.103 (0.82–1.49); *p*:0.520	1.136 (0.86–1.51); *p*:0.376	0.924 (0.77–1.11); *p*:0.409
Ever	38/15	133/36	148/42	281/78	1.00	0.686 (0.34–1.38); *p*:0.291	0.719 (0.36–1.43); *p*:0.346	0.703 (0.37–1.35); *p*:0.285	1.132 (0.71–1.82); *p*:0.607

^a^ The genotyping success rate was 98.81% for rs9787887; ^b^ Adjusted for age, sex, smoking status and alcohol consumption (besides stratified factors accordingly) in a logistic regression model; Fisher exact test was performed when *n* ≤ 5; Bonferroni correction was performed to correct the *p* value, bold values are statistically significant (*p* < 0.0125).

Our analyses demonstrated that neither gender, age, cigarette smoking nor alcohol drinking has detectable impacts on the susceptibility of ESCC after stratified analyses.

### Linkage disequilibrium analyses and association test

**Figure 1:**
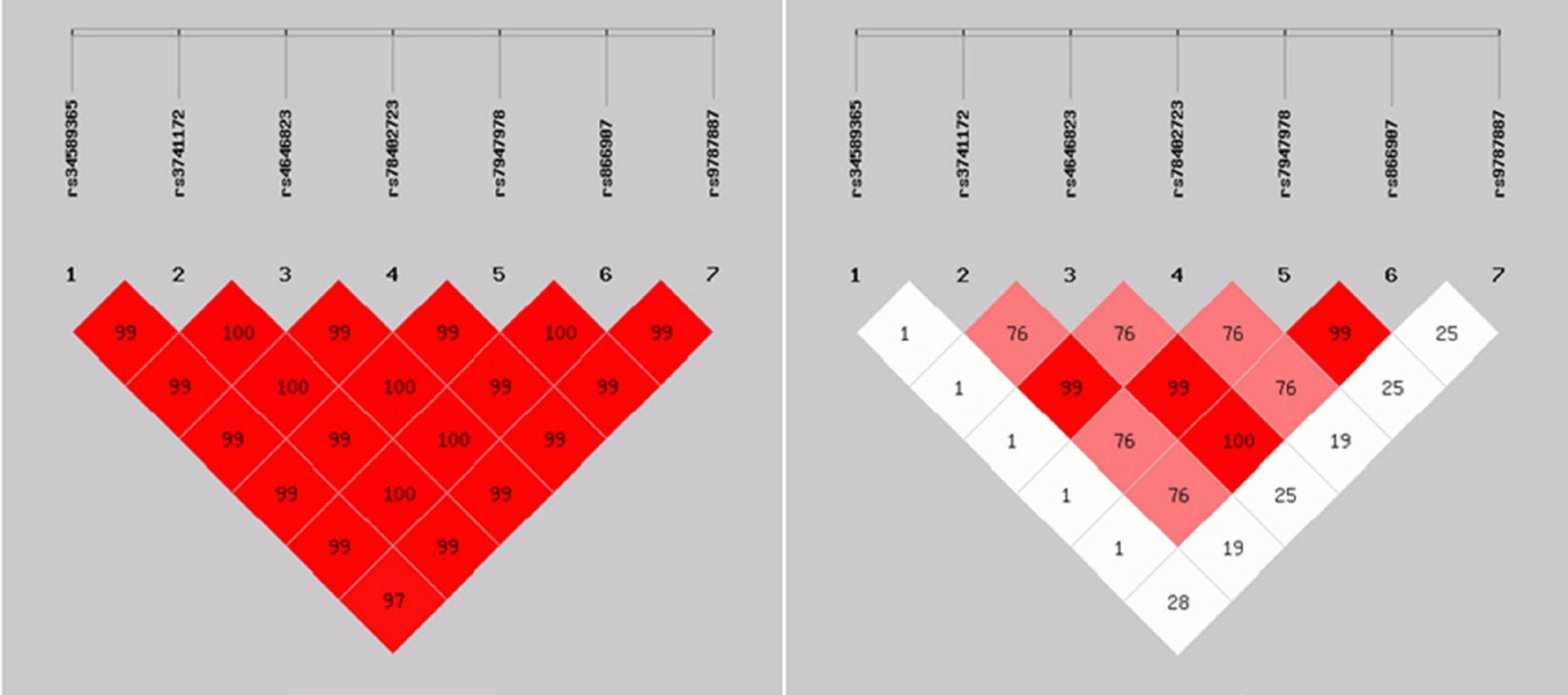
*D*' > 0, *r*^2^ > 0: There were linkage disequilibrium correlations among different loci; *D*' > 70%, *r*^2^ > 30% There were closer linkage disequilibrium correlations among different loci.

**Figure 2:**
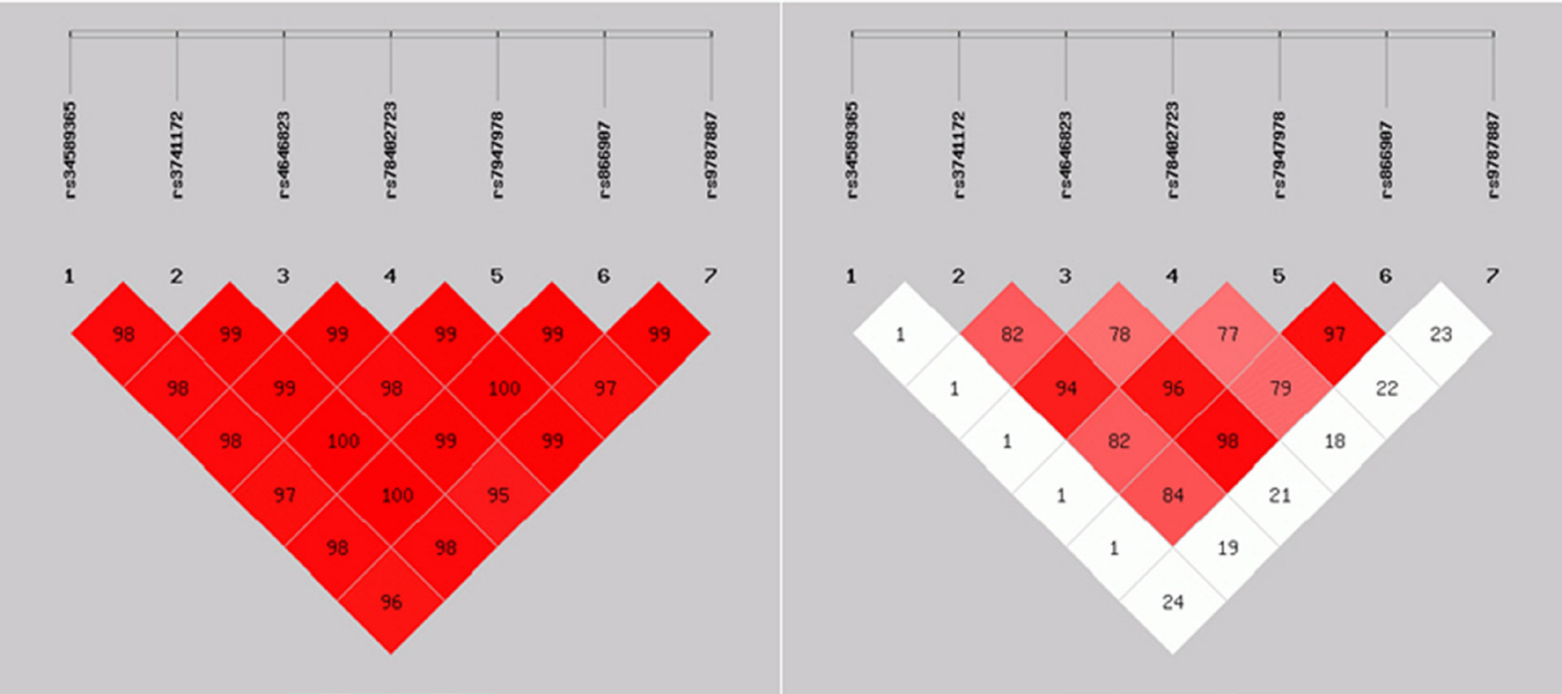
*D*' > 0, *r*^2^ > 0: There were linkage disequilibrium correlations among different loci; *D*' > 70% (0.7), *r*^2^ > 30% (0.3) There were closer linkage disequilibrium correlations among different loci.

Linkage disequilibrium analyses in both controls and cases were performed as shown in Tables 11, 12 and Figures 1, 2. There were strong correlations between these seven loci. Association test was conducted using Haploview software (version 4.2), strong associations were detected between these seven loci.

**Table 11:** Linkage disequilibrium analyses of *ALDH3B2* rs34589365, rs3741172, rs4646823, rs78402723, rs7947978, rs866907 and rs9787887 in control group

*D*’	rs3741172	rs4646823	rs78402723	rs7947978	rs866907	rs9787887
rs34589365	**0.991**	**0.993**	**0.992**	**0.993**	**0.993**	**0.977**
rs3741172	-	1	1	**0.995**	**1**	**1**
rs4646823	-	-	1	**0.996**	**1**	**1**
rs78402723	-	-	-	**0.995**	**1**	**1**
rs7947978	-	-	-	-	**0.996**	**0.993**
rs866907	-	-	-	-	-	**1**
***r*^2^**	**rs3741172**	**rs4646823**	**rs78402723**	**rs7947978**	**rs866907**	**rs9787887**
rs34589365	0.013	0.017	0.013	0.017	0.017	0.272
rs3741172	-	**0.782**	**0.995**	**0.777**	**0.782**	0.182
rs4646823	-	-	**0.786**	**0.988**	**1**	0.233
rs78402723	-	-	-	**0.781**	**0.786**	0.183
rs7947978	-	-	-	-	**0.988**	0.229
rs866907	-	-	-	-	-	0.233

**Table 12:** Linkage disequilibrium analyses of *ALDH3B2* rs34589365, rs3741172, rs4646823, rs78402723, rs7947978, rs866907 and rs9787887 in case group

*D*’	rs3741172	rs4646823	rs78402723	rs7947978	rs866907	rs9787887
rs34589365	**0.984**	**0.982**	**0.983**	**0.979**	**0.981**	**0.969**
rs3741172	**-**	**1**	**1**	**1**	**1**	**0.989**
rs4646823	**-**	**-**	**1**	**0.985**	**1**	**0.957**
rs78402723	**-**	**-**	**-**	**1**	**1**	**1**
rs7947978	**-**	**-**	**-**	**-**	**1**	**0.974**
rs866907	**-**	**-**	**-**	**-**	**-**	**0.991**
**r2**	**rs3741172**	**rs4646823**	**rs78402723**	**rs7947978**	**rs866907**	**rs9787887**
rs34589365	0.014	0.017	0.013	0.017	0.017	0.248
rs3741172	-	**0.828**	**0.943**	**0.824**	**0.845**	0.194
rs4646823	-	**-**	**0.78**	**0.966**	**0.981**	0.219
rs78402723	-	**-**	**-**	**0.776**	**0.796**	0.186
rs7947978	-	**-**	**-**	**-**	**0.976**	0.228
rs866907	-	-	-	-	-	0.231

### Haplotype analyses of ALDH3B2 polymorphisms and susceptibility of ESCC

As demonstrated in Table 13, haplotype analyses showed that ALDH3B2 G_rs34589365_G_rs3741172_T_rs4646823_G_rs78402723_C_rs7947978_G_rs866907_A_rs9787887_ was most common haplotype in both groups (66.3% in controls, 64.6% in cases). As compared with G_rs34589365_G_rs3741172_T_rs4646823_G_rs78402723_C_rs7947978_G_rs866907_A_rs9787887_, none of the haplotypes was associated with the susceptibility of ESCC.

**Table 13:** *ALDH3B2* haplotype frequencies (%) in cases and controls and risk of ESCC

Haplotypes	Case (freq)	Control (freq)	Crude OR(95% CI)	*p*
*ALDH3B2*A_rs34589365_G_rs3741172_T_rs4646823_G_rs78402723_C_rs7947978_G_rs866907_G_rs9787887_	247 (12.1)	323 (12.4)	1.007 [0.840∼1.206]	0.943
*ALDH3B2*G_rs34589365_A_rs3741172_G_rs4646823_A_rs78402723_A_rs7947978_A_rs866907_G_rs9787887_	184 (9.0)	219 (8.4)	1.106 [0.898∼1.363]	0.344
*ALDH3B2*G_rs34589365_G_rs3741172_T_rs4646823_G_rs78402723_C_rs7947978_G_rs866907_A_rs9787887_	1318 (64.6)	1735 (66.3)	1.000	1.000
*ALDH3B2*G_rs34589365_G_rs3741172_T_rs4646823_G_rs78402723_C_rs7947978_G_rs866907_G_rs9787887_	235 (11.5)	279 (10.6)	1.109 [0.919∼1.338]	0.281

Haplotypes were composited by *ALDH3B2* rs34589365, rs3741172, rs4646823, rs78402723, rs7947978, rs866907, rs9787887. All those frequency < 3% were ignored in analysis, most common haplotype *ALDH3B2*G_rs34589365_G_rs3741172_ T_rs4646823_G_rs78402723_C_rs7947978_G_rs866907_A_rs9787887_ was selected as reference.

### Power calculation

The power calculation was performed using the “Power and Sample Size Calculation” software (http://biostat.mc.vanderbilt.edu/wiki/Main/PowerSampleSize). Based on the assumption that the type I error probability for a two sided test α equals 0.05, the probability of exposure in controls P_0_ is 0.126 in control. In the current study, using ligation detection reaction method, the successful rate of genotyping exceeded 98%. There were in total 1315 controls and 1043 cases successfully genotyped. The ratio of control/case (m) equals 1.261, and the correlation coefficient for exposure between matched case and controls (f) is 0.619. The power value is 0.936 as calculated by the “Power and Sample Size Calculation” software.

## DISCUSSION

In this hospital-based case-control epidemiological study, we investigated the association between tagging SNPs of *ALDH3B2* and the risk of developing esophageal squamous cell carcinoma in a Chinese population. We found *ALDH3B2* rs34589365, rs3741172, rs4646823, rs78402723, rs7947978, rs866907 and rs9787887 polymorphisms were not implicated with altered susceptibility of ESCC according to age, gender, cigarette smoking and alcohol drinking stratification analyses.

Despite a suspected association between alcohol drinking and death to cancer reported in an epidemiological study as early as 1903, it took until 1988 for the research community to agree on the potential risk through the International Agency for Research on Cancer (IARC). Clear Patterns have emerged between alcohol consumption and esophageal cancer. Essentially, alcohol and its metabolite acetaldehyde were both designated as type 1A carcinogen [13]. The cytotoxic properties, the ability to form DNA-acetaldehyde adducts and to generate additional mutagenic species at concentrations attainable *in vivo* may underlie the carcinogenic effects [22]. Most of the acetaldehyde generated during alcohol metabolism *in vivo* is rapidly eliminated by aldehyde dehydrogenase (ALDH), which renders ALDH an important role in carcinogen balancing and therefore carcinogenesis. In fact, there is ample evidence showed that subjects with an inactive form of ALDH2 (heterozygous for ALDH2 mutation) have an increased risk of developing various types of head and neck cancers as a consequent of intense exposure to acetaldehyde. Case-control studies of various Japanese drinking populations [23–29] and Chinese alcoholics [30] have consistently reported that the inactive ALDH2 encoded by the ALDH2*1/2*2 genotype is a strong risk factor for esophageal cancer. In resemblance with ALDH2, ALDH3 also plays a pivotal role in the alcohol metabolism, we thus hypothesize that ALDH3 family may be of potential relevance to carcinogenesis. In line with our speculation, overexpression of ALDH3 protects cells from 4-hydroxynonenal induced apoptosis, suggesting a functional relevance of ALDH3 in the carcinogenesis. On the other hand, many of the sphere cells and stem cells reported in different organs have recently been found to be associated with elevated ALDH1A1 enzyme activity [31–33]. ALDH1A1 expression or activity may be used with other cell surface markers to identify tumor-initiating cells in hepatocellular, prostate and breast solid carcinomas [34–36]. ALDH1A1 has also been detected to be associated with early metastasis and poor clinical outcome [33]. In addition, the ALDH enzymes also play a pivotal role in epithelial homeostasis. Deregulation of these enzymes is associated with multiple cancers [37–41]. In the current study, we demonstrated that there are no significant association between ALDH3B2 SNPs and risk of ESCC for the first time. More importantly, we have analyzed the interaction of the genetic background and environmental risk factors. These findings indicate that ALDH3B2 may not be the primary contributor to ESCC carcinogenesis. Considering the biological function of ALDH3B2, the evaluation with combined considerations based on both genetic and environmental factors would be much more precise and meaningful.

Cigarette smoking and alcohol drinking have emerged as well-known risk factors of ESCC. Although *ALDH3B2* rs34589365, rs3741172, rs4646823, rs78402723, rs7947978, rs866907 and rs9787887 were not associated with the susceptibility of ESCC in the current study, the cigarette smoking rate and alcohol drinking rate were significantly higher in the ESCC cases, exemplifying the significance of interaction between the environmental and genetic risk factors in causing esophageal squamous carcinoma.

Our finding that there are more male than female subjects in the case group was in consistent with the comprehensive data recently published by the National Office for Cancer Prevention and Control as well as the National Cancer Center of China. The fact that smoking and alcohol drinking are far more prevalent in male subjects implicated the importance of these risk factors in carcinogenesis of ESCC.

One of the limitations of our previous studies investigating the association between SNPs and risk of ESCC was the sample size [12]. To overcome that, we have recruited a total of 2358 subjects including 1043 ESCC cases and 1315 controls in the current study. Yet, we do acknowledge there are still some limitations in this study. First, this study is limited by the sample sources, future studies designed and conduced in multiple ethnical populations and various geographic locations would be more convincing. Second, the lack of a validation cohort compromised the power of our study. Third, we are refrained by the lack of technical support to establish single nucleotide mutation cell/animal model, the actual biological function of these SNPs in esophageal carcinoma remains obscure, the underlying mechanisms are yet to be further dissected. Last but not least, the detailed information with regard to cancer metastasis and survival were not provided, the correlations between SNPs and outcomes have not been analyzed as this follow-up is still ongoing.

## MATERIALS AND METHODS

### Ethics statement

This hospital-based case-control study was approved by the Review Board of Jiangsu University (Zhenjiang, China). All subjects provided written informed consents. This study has complied with the World Medical Association Declaration of Helsinki with regard to ethical conduct of research involving human subjects and/or animals.

### Study populations

A total of subjects consisting of 1043 ESCC cases and 1315 non-cancer controls frequency-matched to the cases regarding age and gender (*p* = 0.121 and 0.880, respectively) were enrolled in this study (Table 1). All patients and controls were consecutively recruited from the Affiliated People's Hospital of Jiangsu University (Zhenjiang, China) from October 2008 to January 2017. All cases of esophageal cancer were diagnosed as ESCC histologically. The exclusion criteria included cancer history, metastasized cancer or chemotherapy/radiotherapy history.

Each subject was individually questioned by experienced interviewers with a questionnaire to obtain information on demographic information and related risk factors (including alcohol consumption and cigarette smoking). After written informed consent was provided, two milliliters of venous blood were collected from each subject. The “Smokers” cohort included individuals who smoked one cigarette per day for more than one year. Subjects who had more than three alcoholic drinks a week for more than six months were included in the “Alcohol drinkers” cohort.

### Genomic DNA extraction, SNP selection and genotyping

Genomic DNA was isolated from peripheral blood using QIAamp DNA Blood Mini Kit (Qiagen, Berlin, Germany) as reported [12]. Sample DNA were amplified by PCR according to the manufacturer's protocol. Gene polymorphisms were analyzed by the ligation detection reaction (LDR) method with technical support from Genesky Biotechnology Inc. (Shanghai, China). 10% of the total samples were randomly selected for repeated analyses for quality control. Pilot linkage disequilibrium analyses were performed in the Chinese Han population to choose the SNP loci with moderate correlation, and tag SNPs were selected for further analyses.

### Statistical analyses

Statistical analyses were conducted using SPSS 23.0 statistical package (SPCC Inc., Chicago, IL). Hardy-Weinberg equilibrium for genotypes was tested by goodness-of-fit χ^2^ in control group. Variations of demographic characteristics and genotypes of the *ALDH3B2* rs34589365, rs3741172, rs4646823, rs78402723, rs7947978, rs866907 and rs9787887 between the controls and cases were evaluated using the *chi*-square (χ^2^) test to examine the statistical differences. The associations between these seven SNPs and risk of ESCC were analyzed by PLINK software (v1.07, available at http://zzz.bwh.harvard.edu/plink/download.shtml). Crude ORs and adjusted ORs when adjusting for age, sex, smoking and alcohol drinking status were also computed using logistic regression analyses. Bilateral probability tests were taken, *p* value < 0.05 was considered statistically significant.

## CONCLUSIONS

The esophageal squamous carcinoma is associated with various factors including gene, environment and life-style. Our findings that *ALDH3B2* rs34589365, rs3741172, rs4646823, rs78402723, rs7947978, rs866907 and rs9787887 polymorphisms were not implicated with altered susceptibility of ESCC in different age, gender, cigarette smoking and alcohol drinking status, when interpreted with caution, could be helpful in evaluating the susceptibility to ESCC.
